# A decrease in retinal progenitor cells is associated with early features of diabetic retinopathy in a model that combines diabetes and hypertension

**Published:** 2008-09-11

**Authors:** Jacqueline Mendonça Lopes de Faria, Kamila Cristina Silva, Patrícia Aline Boer, Tiago Correa Cavalcanti, Mariana Aparecida Brunini Rosales, Ana Luiza Ferrari, José Butori Lopes de Faria

**Affiliations:** Renal Pathophysiology Laboratory, Investigation in Diabetes Complications, Department of Internal Medicine, Faculty of Medical Sciences, State University of Campinas, Campinas, São Paulo, Brazil

## Abstract

**Purpose:**

Hyperglycemia and hypertension contribute to the development of diabetic retinopathy, and this may involve alterations in the normal retinal cell cycle. In this work, we examined the influence of diabetes and hypertension on retinal cell replication in vivo and the relationship between these changes and several early markers of diabetic retinopathy.

**Methods:**

Diabetes was induced with streptozotocin in 4- and 12-week-old spontaneously hypertensive rats (SHR) and their Wistar Kyoto (WKY) controls. The rats were killed 15 days later. Retinal cells stained with bromodeoxyuridine (BrdU) were seen in rats of both ages.

**Results:**

In 12-week-old rats, the number of BrdU-positive retinal cells was higher in SHR than in WKY rats. After 15 days of diabetes mellitus, there was a marked reduction in cell replication only in diabetic SHR (p=0.007). The BrdU-positive cells expressed neural, glial, or vascular progenitor markers. There was greater expression of p27^Kip1^ in the ganglion cell layer of both diabetic groups (p=0.05), whereas in the inner nuclear layer there was enhanced expression only in diabetic SHR (p=0.02). There was a marked increase in the retinal expression of fibronectin (p=0.04) and vascular endothelial growth factor (p=0.02) in diabetic SHR that was accompanied by blood-retinal barrier breakdown (p=0.01).

**Discussion:**

Concomitant diabetes and hypertension attenuated the proliferation of retinal cells, and it is associated with an increase in p27^Kip1^ expression, fibronectin accumulation, and blood-retinal barrier breakdown. The replicative retinal cells displayed characteristics of progenitor cells.

## Introduction

Diabetic retinopathy is a progressive neurologic disease that is characterized by neuronal degeneration and extensive vascular changes. However, our knowledge of the mechanisms leading to neuronal cell loss and vascular dysfunction in diabetic retinopathy is still incomplete. We do know cells other than endothelial cells and pericytes are affected by hyperglycemia in diabetes [1,2]. Clinical studies [3-8] have shown that hyperglycemia causes neural dysfunction in the retina before the onset of diabetic microvasculopathy.

The development of retinal disease varies among patients with diabetes. Although hypertension may be an important contributor, the precise mechanism by which hypertension can exacerbate diabetic retinopathy remains to be established. Diabetic individuals frequently have concomitant retinopathy and nephropathy, and it has been suggested that similar mechanisms may be involved in these two long-term complications of diabetes [9]. Inhibitors of cyclin-dependent kinases (Cdk) such as p27^Kip1^, a negative cell cycle regulator, are involved in the development of diabetic nephropathy, with associated mesangial hypertrophy and extracellular matrix accumulation [10-13]. In addition, genetic hypertension potentiates the cell-cycle abnormalities induced by renal hyperglycemia [14]. These findings suggest that cell cycle regulators are altered by the diabetic milieu and that such alterations contribute to the pathogenesis of diabetic microvascular complications.

The early phase of diabetic retinopathy involves microangiopathy characterized by a diffuse increase in vascular permeability and capillary basement membrane thickening [15-18]. Fibronectin, a component of the basement membrane, is overexpressed in the retina of diabetic adults [15]. Experimental studies have indicated that this accumulation of fibronectin in retinal tissue is simply an epiphenomenon of the diabetic state, but may be operative in sight-threatening diabetic retinopathy. Indeed, the downregulation of fibronectin production in galactose-fed rats partly prevented retinal basement membrane thickening and reduced pericyte and endothelial cell loss [19].

The developing and postnatal vertebrate retina contains neural progenitor cells that divide, generate neurospheres, and undergo neuronal and glial differentiation [20-23]. These cells can be identified by their ability to proliferate based on the incorporation of bromodeoxyuridine (BrdU), and by the expression of progenitor markers such as nestin, membrane receptor tyrosine kinase, also designated vascular endothelial growth factor receptor-2 (Flk-1), and paired box gene 6 (Pax6) [24-27]. p27^Kip1^ has recently been implicated in the molecular mechanism that controls the decision of multipotent central nervous system progenitors to withdraw from the cell cycle and to maintain the differentiated state of the postmitotic cell [28]. In the retina, p27^Kip1^ is expressed in a pattern coincident with the onset of the differentiation of most retinal cell types, and in vitro the accumulation of p27^Kip1^ in retinal cells correlates with cell cycle withdrawal and differentiation, thereby inhibiting progenitor cell proliferation [28].

These observations prompted us to search for potential progenitor cells in adult rat retina. We also wanted to assess the impact of hypertension and short-term diabetes on the number of retinal BrdU positive cells and to examine the relationship between these cells and the well known abnormalities associated with the early stages of diabetic retinopathy. We have identified a small population of potential progenitor cells characterized by BrdU positivity that colocalize with nestin, protein kinase C-alpha (PKC-α), Flk-1, and glial fibrillary acidic protein (GFAP) antigens. Furthermore, the higher number of 84 proliferating cells in the retina of adult spontaneously hypertensive rats compared with normotensive rats was 85 significantly reduced in diabetic SHR rats. These findings were associated with enhanced expression of p27^Kip1^ and with the classic abnormalities of diabetic retina—i.e., fibronectin accumulation, increased expression of retinal vascular endothelial growth factor (VEGF), and blood-retinal barrier breakdown.

## Methods

The experiments were done in accordance with the ARVO Statement for the Use of Animals in Ophthalmic and Vision Research. The protocol for this study complies with the guidelines of the Brazilian College for Animal Experimentation (COBEA) and was approved by the institutional Committee for Ethics in Animal Research (CEEA/IB/UNICAMP, protocol no. 1408–1). This study used spontaneously hypertensive rats (SHR) and their genetically normotensive counterparts, Wistar Kyoto rats (WKY), which were derived from animals supplied by Taconic (Germantown, NY) and bred in our animal facility. The rats were housed at a constant temperature (24 °C) on a 12 h:12 h light-dark cycle with access to food and tap water ad libitum. The rats were weighed on the day before the induction of diabetes and before they were euthanized.

Experimental diabetes was induced in 4- and 12-week-old hypertensive male SHR and WKY rats, who had been allowed to fast overnight. Each animal was given a single intravenous injection of 50 mg/kg streptozotocin (STZ; Sigma, St. Louis, MO), dissolved in sodium citrate buffer, pH 4.5, within 5 min of its preparation. Control rats received only vehicle (500 μl citrate buffer). Blood glucose levels were measured using an enzymatic colorimetric GOD-PAP assay (Merck, Darmstadt, Germany) 72 h after the injection of STZ or citrate buffer and on the day before euthanasia. Values ≥15 mmol/l were indicative of diabetes. Systolic blood pressure was obtained by tail-cuff plethysmography (Physiograph^®^ MK-III-S; Narco Bio-System, Houston, TX) in unanesthetized, warmed rats. Three to five determinations were made per rat. Readings were taken on the day before the induction of diabetes and before rats were euthanized. Rats were habituated with the procedure for measurement before taking the blood pressure readings.

Fifteen days after the induction of diabetes, retinal capillary permeability was assessed, and the rats were euthanized with an overdose (twice the anesthesic dose) of anesthesia. The eye globes and retinas were collected for immunohistochemistry, immunofluorescence, and western blot analyses. We have previously described early inflammatory and oxidative changes in retina from diabetic SHR rats after 20 days of diabetes mellitus [29,30]. The aim of the present work was to examine the alterations in cellular proliferation and apoptosis that precede the changes we have mentioned, and to investigate the relationship between these changes and the well established abnormalities associated with the early stages of diabetic retinopathy.

### Detection of proliferating cells in vivo

Retinal cell replication was evaluated based on the incorporation of BrdU (Calbiochem, La Jolla, CA), a thymidine analog that incorporates into DNA in the S phase [14,31]. Briefly, the rats were given an intraperitoneal injection of 100 mg/kg BrdU, dissolved in saline, 1 h before they were euthanized. An equal number of control rats received saline alone. The rats were euthanized with an intraperitoneal injection of 30 mg/kg sodium pentobarbital (Hipnol, Fontoveter, Itapira, SP, Brazil). Their eyes were enucleated, and 130 a portion of the gastrointestinal tract (positive control for BrdU staining) were excised. The eye globe and the GI tract were 131 fixed in buffered formalin, and embedded in paraffin-embedded, or placed in OCT 132 cryoprotector (Tissue-Tech, Sakura, CA) and snap frozen in liquid nitrogen for subsequent sectioning in a cryostat. Next, 4 µm-thick consecutive sections were mounted on silane-coated slides for immunofluorescence.

### Immunohistochemistry for BrdU and p27^Kip1^

Detection of BrdU and p27^Kip1^ was performed by dewaxing slides of eye sections, rehydrating them, and placing in 2 N HCl at 31 °C for 20 min and in 0.005% trypsin in phosphate-buffered saline (PBS) at 37 °C for 2 min for antigen retrieval. After this pretreatment, the slides were placed in 1% nonfat milk in PBS for 1 h to block nonspecific sites. The sections were then incubated with a 1:50 dilution of mouse antihuman antibodies for BrdU (Dako, Glostrup, Denmark) or a 1:100 dilution of mouse monoclonal anti-p27^Kip1^ antibody (Transduction Laboratories, Lexington, KY) for 1.5 h at room temperature. The slides of eye sections were washed in, a biotinylated secondary antimouse IgG antibody (Vector, Burlingame, CA) was applied and allowed to sit for 1 h. Endogenous peroxidase was blocked by incubating the slides in 3% H_2_O_2_ for 5 min. To detect p27^Kip1^, we performed microwave post-fixation of slides immersed in 0.01 M citrate buffer, pH 6.0 using a domestic oven (Panasonic Junior, Sao Paulo, SP, Brazil) operated at 700 W. The slides were incubated with an avidin-biotin complex (ABC) reagent (Vector) for 30 min followed by the addition of diaminobenzidine tetrahydrochloride (DAB; Sigma) as a substrate-chromogen solution. Next, the slides were counterstained with hematoxylin before they were dehydrated and mounted in Entellan (Merck, Darmstadt, Germany). Positive controls for BrdU staining consisted of sections of the gastrointestinal tract of each rat. Negative controls for the reaction consisted of omitting the primary antibody.

Quantitative analysis of BrdU positivity was done by an observer unaware of the slide identification. The results were expressed as the total number of positive cells counted in eight random retinal sections from the right eye of each rat. For p27^Kip1^, the results were expressed as a percentage of positive cells in the ganglion cell and inner nuclear layers, using the following scale: 0 (no positivity), 0.5 (up to 10% positivity), 1 (11%–25% positivity), 1.5 (26%–40% positivity), 2 (41%–53% positivity), 2.5 (54%–66% positivity), 3 (67%–80% positivity), and 4 (>80% positivity) [29]. The intervening distance between retinal sections was approximately 24 μm.

### Immunocolocalization by immunofluorescence

The slides were fixed with acetone for 3 min at room temperature, after which they were washed with PBS, pH 7.4, and blocked in PBS containing 5% bovine serum albumin (BSA) for 2 h at room temperature. The sections were then incubated with the appropriate primary antibody to identify glial, endothelial, and neuronal cells (1:10 goat polyclonal anti-GFAP antibody; Santa Cruz Biochemical, Santa Cruz, CA; 1:100 mouse monoclonal anti-FLK-1 antibody; Santa Cruz; 1:10 mouse antinestin antibody; BD PharMingen™, Franklin Lakes, NJ; and 1:10 mouse monoclonal anti PKC-α antibody; Abcam, Inc., Cambridge, MA) for 5 h at room temperature. After they were washed with PBS, the sections were incubated with secondary antibody (donkey antigoat IgG-FITC or goat antimouse IgG-FITC) as appropriate for 1 h at room temperature. The slides were then washed with PBS and incubated with 1:10 sheep polyclonal anti-BrdU antibody (Abcam, Inc.) overnight at 4 °C, followed by another wash with PBS and incubation with rabbit polyclonal antibody to 1:1,000 sheep IgG-rhodamine (1,5 mg of IgG; Abcam) for 1 h at room temperature. Finally, the sections were rinsed with PBS then coverslipped with Vectashield antifading medium containing 4',6'-diamino-2-phenylindole (DAPI) to stain nuclei (Vector). The sections were examined under a confocal laser scanning microscope (CLSM, LSM510 Zeiss, Jena, Germany) with appropriate emission filters for FITC and rhodamine. Digital images were captured using specific software (LSM; Zeiss). The negative controls consisted of omitting the primary antibody.

### Terminal deoxynucleotidyl transferase-mediated nick-end labeling

To determine whether retinal cell apoptosis was influenced by age, diabetes or rat strain, the terminal deoxynucleotidyl transferase (TdT)-mediated dUTP-biotin nick-end labeling (TUNEL) method for detecting DNA breaks in situ [32] was applied to retinal tissue from the same rats used for BrdU immunostaining. [14,32]. Sections (4 μm thick) were deparaffinized, boiled in 0.01 M citric acid, pH 6.0, and incubated with 9.3 µg/ml proteinase K (Boehringer Mannheim, Indianapolis, IN) for 15 min at room temperature. Endogenous peroxidase was quenched, and sections were rinsed in One-Phor-All buffer (Amersham Pharmacia Biotech, Piscataway, NJ), pH 7.2, and incubated with diluted 1:50 TdT (Amersham Pharmacia) and diluted 1:50 biotinylated-dUTP (Gibco, Grand Island, NY) in TdT buffer (100 mM TRIS, 1 mM dithiothreitol, 50% of glycerol, 0.1% sodium azide, 0.01%, Brij®35 [Polyoxyethyleneglycol dodecyl ether], pH 7.2) for 60 min at room temperature. Labeled nuclei were detected with ABC Vectastain (Vector) in PBS and DAB, chloride, and hydrogen peroxide and counterstained with hematoxylin. As a positive control, some slides were treated with 20 Kunitz units/ml DNase (Sigma). The quantitative analysis for TUNEL-positive cells was done by an observer with no knowledge of the studied groups. Results were expressed as the number of positive cells per retinal section in at least eight random retinal sections from the right eye of each animal. The distance between sections was approximately 24 μm.

### Isolation of retina

The eyes were enucleated, and the retinas were dissected and isolated from the retinal pigmented epithelium. The retinas were lysed directly on ice in 300 µl of a buffer containing 2% SDS and 60 mM Tris-HCl (pH 6.8) supplemented with a cocktail of protease inhibitors (Complete^®^, containing irreversible and reversible protease inhibitors such as antipain, aprotinin, bestatin, chymostatin, Ethylenediaminetetraacetic acid (EDTA), leupeptin, pepstatin and phosphoramidon; Boehringer-Mannheim). The lysed retinas were centrifuged. The supernatants were transferred to new tubes, and the protein concentrations were measured by the Bradford method [33], using BSA as the standard.

### Western blotting for fibronectin, GFAP, and VEGF

For western blotting, 30 µg (for GFAP), 50 µg (for fibronectin), and 100 µg (for VEGF) of total retinal protein in 5% glycerol/0.03% bromophenol blue/10 mM dithiothreitol was loaded onto 8%–15% SDS polyacrylamide gels. Molecular weight markers (Rainbow; Amersham Pharmacia) were used as standards. After electrophoresis, proteins were transferred to nitrocellulose membranes (Bio-Rad, Hercules, CA) in transfer buffer consisting of 50 mM Tris-HCl, pH 7.0, 380 mM glycine, 0.1% SDS, and 20% methanol. Nonspecific binding was blocked by incubating the membranes overnight at 4 °C in 5% nonfat milk, or at 4 °C in PBS containing 1% gelatin with 0.1% Tween 20 (Fisher Chemicals, Fairlawn, NJ) for VEGF. The membranes were then incubated for 1 h at room temperature with primary antibodies: 1:1,000 goat monoclonal anti-fibronectin antibody (Calbiochem, San Diego, CA), 1:100 polyclonal goat-GFAP antibody (Santa Cruz, Santa Cruz, CA), and 1:5000 rabbit polyclonal IgG anti-VEGF antibody (Santa Cruz). The blots were subsequently washed in Tris-buffered saline with Tween and incubated with horseradish peroxidase (HRP)-conjugated secondary antibody (New England Biolabs, Beverly, MA). Immunoreactive bands were visualized with the enhanced chemiluminescence method (Super Signal CL-HRP Substrate System; Pierce, Rockford, IL). Exposed films were scanned with a densitometer (Bio-Rad) and analyzed quantitatively with Multi-Analyst Macintosh Software for Image Analysis Systems Bio Rad (Hercules, CA). Western blots were repeated 3–5 times; qualitatively similar results were obtained each time. Equal loading and transfer was ensured by reprobing the membranes for β-actin.

### Quantitative measurement of retinal capillary permeability using Evans blue dye

Briefly [34], the rats (SHR and WKY) were anesthetized with an intraperitoneal injection of 30 mg/kg sodium pentobarbital, and the left femoral vein and right femoral artery were cannulated with 0.28 mm internal diameter polyethylene tubing (Becton Dickinson, Sparks, MD) filled with heparinized saline (400 units heparin/ml saline). Next, 45 mg/kg Evans blue dye was injected through the femoral vein over a period of 10 s. The blue color of the rats confirmed the uptake and distribution of the dye. Two minutes after the injection of the dye, 0.2 ml of blood was drawn from the femoral artery to determine the initial Evans blue plasma concentration. Subsequently, 0.1 ml of blood was drawn from the femoral artery at 15 min intervals for up to 2 h postinjection to obtain the time-averaged Evans blue plasma concentration. Exactly 2 h after infusion, needle was inserted into the left ventricle and the rats were perfused for 2 minutes at 37°C with 0.05 M, pH 3.5, citrate-buffered paraformaldehyde at a physiological pressure of 120 mm Hg.

Immediately after perfusion, both eyes were enucleated and bisected at the equator. These animals were euthanized with an injection of overdose of penthabarbitol. The retinas were carefully dissected under an operating microscope and weighed. They were then dried in a Speed-Vac for approximately 5 h. The Evans blue dye was extracted from the tissue by incubating each retina in 120 µl formamide (Sigma) for 18 h at 70 °C followed by centrifugation at 27,000x g for 45 min in a Beckman TLX rotor at 4 °C. The absorbance of 60 μl aliquots of the supernatant was measured spectrophotometrically in triplicate at A_620 nm_ at 5 s intervals. The absorbances were corrected for background readings, and the dye content of the extracts was calculated from a standard curve of Evans blue in formamide. Blood-retinal barrier breakdown was calculated using the following equation:

(µg of Evans blue)( time-averaged µg of Evans blue)/(retinal wet weight in g)(µl of plasma x h of circulation)

The results were expressed as µl of plasma x retinal wet wt^−1^ (g) x h^−1^.

### Statistical analysis

The results were expressed as the means±standard deviation or standard error of measurement as indicated. Comparisons between groups were done using ANOVA followed by Fisher’s protected least-significant difference test. All comparisons were done using the StatView statistics software for Macintosh, with a value of p<0.05 indicating significance.

## Results

SHR rats are normotensive when they are 4 weeks old, but are fully hypertensive by the time they are 12 weeks old. We used both age groups because they allowed us to assess the contribution of the genetics of hypertension (4-week-old rats) and the influence of genetics plus hypertension per se (12-week-old rats) on retinal abnormalities. As previously demonstrated by our group and others, the systolic blood pressure (SBP) in 4-week-old SHR was significantly higher than in WKY, although still within the normal range (Table 1). The SBP of 12-week-old SHR was significantly higher (p=0.0001) than that of age-matched WKY (Table 1). Bodyweight was lower, and blood glucose levels were higher in diabetic rats than in their respective controls at both ages (p=0.0001; Table 1).

**Table 1:** General characteristics of the studied rats

**Parameter**	**Younger rat groups**				**Adult rat groups**			
	**CT-WKY**	**DM-WKY**	**CT-SHR**	**DM-SHR**	**CT-WKY**	**DM-WKY**	**CT-SHR**	**DM-SHR**
N	20	24	25	24	22	26	29	26
Final body weight (g)	198±21	139±23†	134±15	105±12†	406± 54	293±43†	267±42	189±25†
Systolic blood pressure (mmHg)	140 ±7‡	142 ±5‡	118±13	120±13	174±14‡	174±18‡	117±12	115±14
Glycemia (mmol/l)	6.9±0.3	30.4±2.5§	7.1±0.5	28.6±4§	6.2±1.3	25.6±5.2§	5.8±1.6	25.3±4.2§

The following abbreviations are in effect: CT-WKY: control WKY; DM-WKY: diabetic WKY; CT-SHR: control SHR; DM-SHR: diabetic SHR; SBP: systolic blood pressure; * p<0.005 versus respectively WKY groups, ^†^ p<0.003 versus control groups, ^‡^ p=0.0001 versus WKY groups, ^§^ p<0.0001 versus control groups.

### Cells with progenitor characteristics in adult rat retina

**Figure 1:**
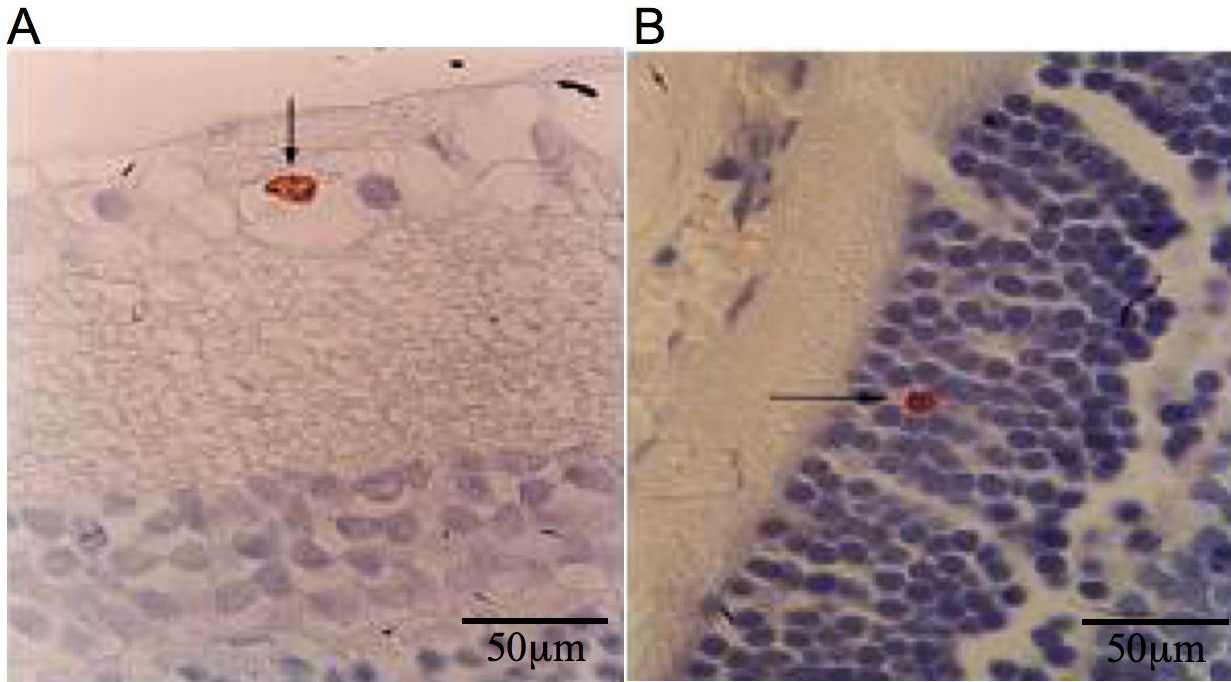
Immunohystochemistry for BrDU in retinal slides from studied rats. The BrDU positive cells, although rare, are localized in ganglion cell layer **(A)** and in inner nuclear layer **(B).** The scale bar represents 50 µm.

**Figure 2:**
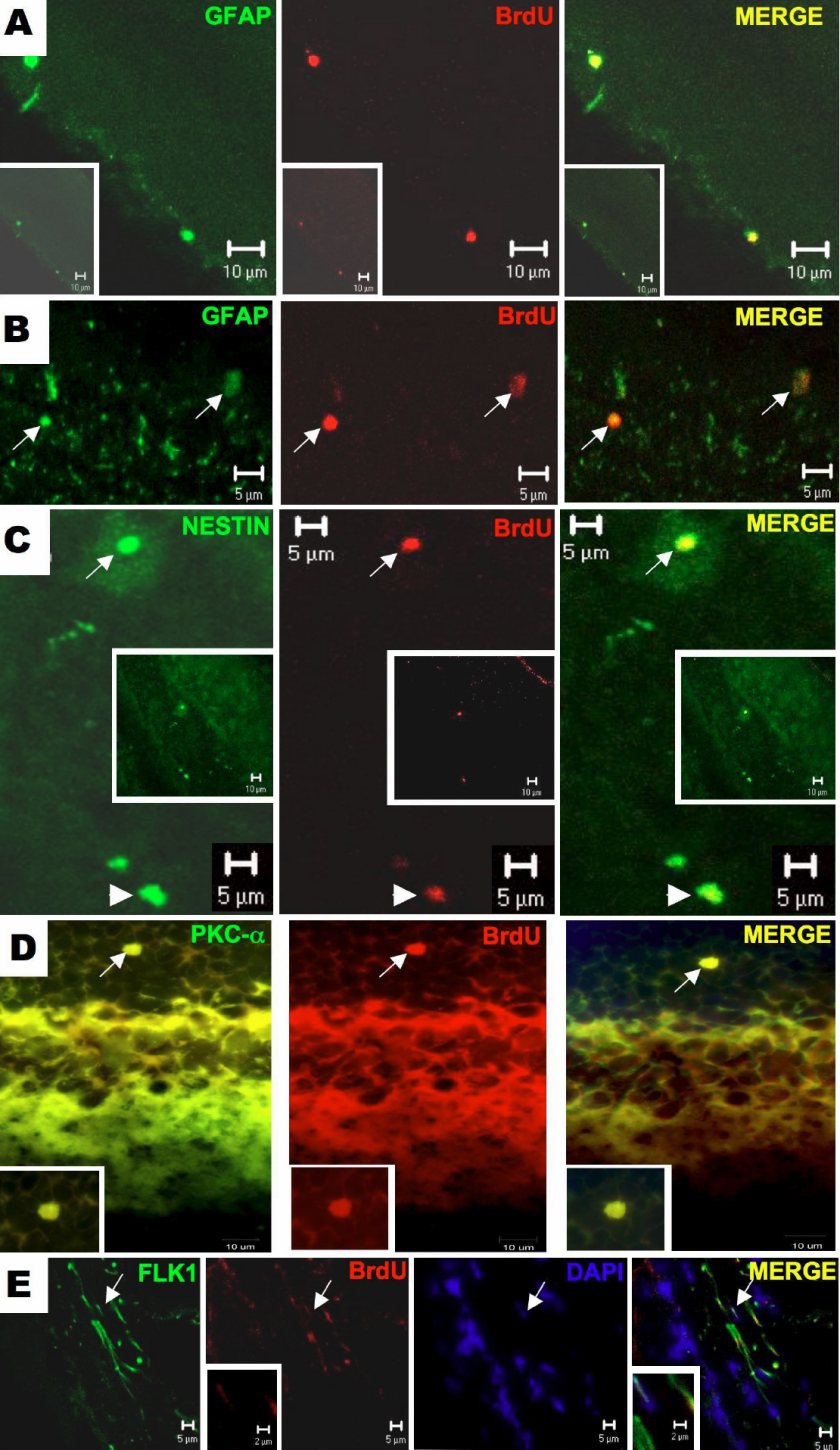
Immunofuorescence assay for double labeling of BrDU positive cells in retinal sections against glial, neuron and endothelial markers. In panel **A**, it is shown a retinal section showing two cells labeled for glial fibrillar acidic protein (GFAP) and BrdU in the ganglion cell layer. In **B**, there are the presence of two BrdU-positive cells in outer nuclear layer of the retina co-stained with GFAP antibody. In this slide (**C**), the BrDU positive cell is stained for nestin in the inner nuclear (long arrows) and ganglion cell (short arrows) layers of the retina. For identification of amacrine/bipolar origin of BrDu positive cell in the retina, an immunofluorescence for PKC- α antigen was performed. There is a BrDU positive cell that also stained for PKC- α in the outer nuclear (**D**). In panel E, elongated endothelial cells were identified expressing both Flk-1 and BrdU, localized in theinner nuclear layer of the retina

BrdU-positive-stained cells were rare but clearly identifiable in retinal sections of adult rats (Figure 1A,B). The proliferating retinal cells of adult rats were characterized further by using specific cell markers, including GFAP, the tyrosine-kinase receptor Flk1, the intermediate filament protein nestin, and PKC-α. BrdU and GFAP colocalized in cells of the ganglion cell layer, suggesting that they may represent glial cells in proliferation (Figure 2A,B). Other BrdU-positive cells were also colabeled with nestin (Figure 2C), an intermediate filament structural protein expressed in primitive neural tissue. The detection of this protein in the inner nuclear and ganglion cell layers suggested the presence of neural progenitor cells. Figure 2D shows a PKC-α-positive cell in the outer nuclear layer that was also positive for BrdU. In this case, cellular maturation involved a bipolar–amacrine neural cell. The elongated BrdU-positive cells in the inner nuclear layer reacted strongly with antibody against Flk-1 (Figure 2E), a surface receptor protein characteristic of endothelial cells in cell cycle progression. These findings indicate that adult rat retina contains different populations of replicating cells with characteristics of glial or endothelial cells. The detection of cells that coexpressed BrdU and nestin or PKC-α suggests that subpopulations of replicating cells may differentiate into neural cell subtypes.

### Diabetes decreases the number of progenitor cells only in retina from 12-week-old SHR

**Figure 3:**
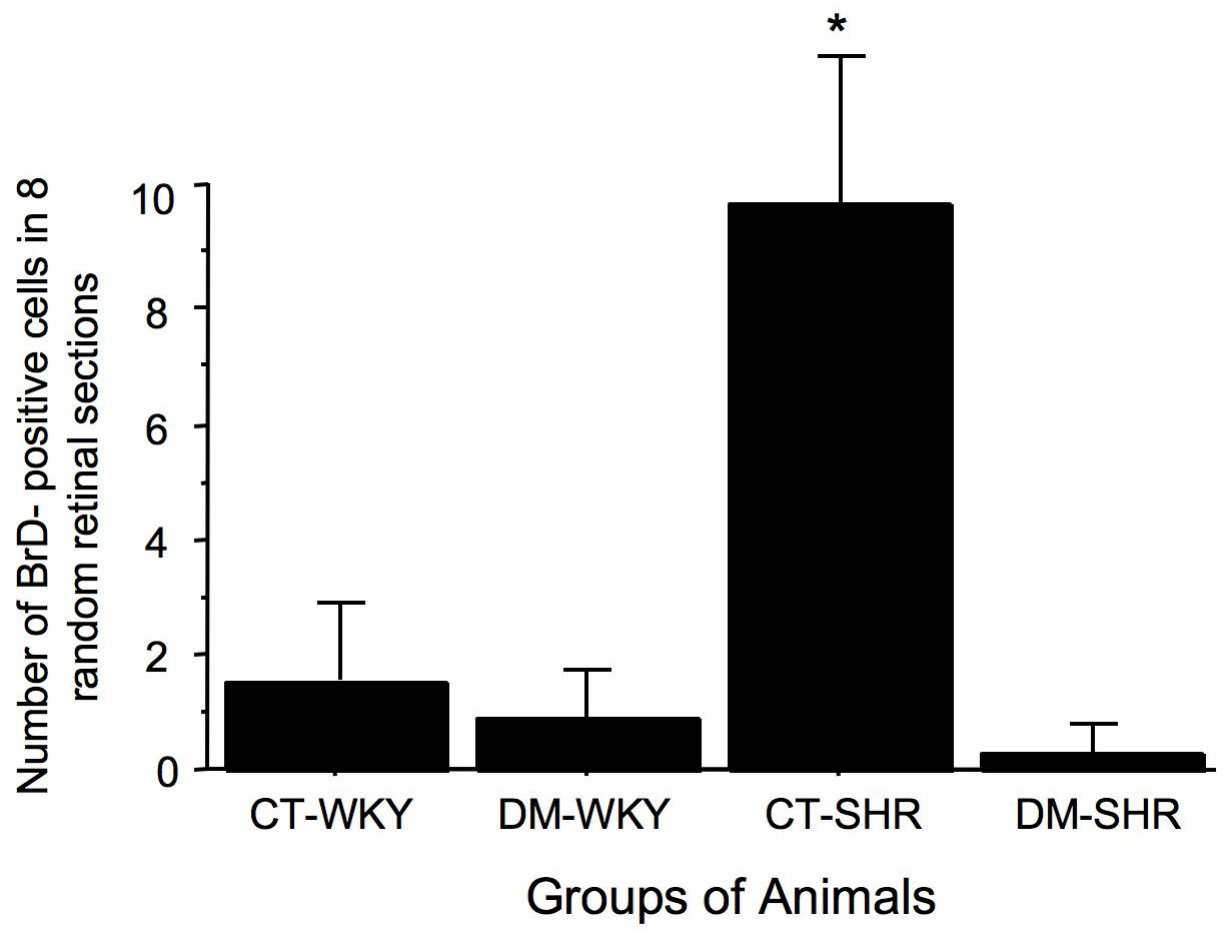
Total number of BrdU-positive cells counted in eight random retinal sections from 12-week-old rats. See Methods for further details. The results are expressed as the mean±SEM; asterisk (*) is p=0.02 versus CT- spontaneously hypertensive rats (SHR). The number of BrDU positive cells present in retina from WKY rats is very low and do not change according to experiment condition (presence of experimental diabetes); by contrast in SHR rats, it is observed a higher number of these BrDU cells in retina and the induction of diabetes dramatically reduced this number abbreviations used are as follows: control WKY (CT-WKY), diabetic WKY (DM-WKY), control SHR (CT-SHR), and diabetic SHR (DM-SHR).

The total number of BrdU-positive retinal cells in 12-week-old rats was significantly higher in hypertensive SHR than in normotensive WKY (9.5±4.0 versus 1.2±0.4 positive retinal cells; p=0.01). After 15 days of diabetes mellitus, there was a significant decrease in the number of these cells only in SHR (0.2±0.2 positive retinal cells; p=0.007; Figure 3). In contrast, in 4-week-old SHR and WKY rats, the number of proliferating cells was similar in all groups (4.5±0.5 for control WKY versus 2.8±0.4 for diabetic WKY versus 3.4±0.4 for control SHR versus 4.0±0.5 for diabetic SHR BrdU-positive cells (p>0.05).

### TUNEL staining is unaltered by rat strain, animal age, or short-term diabetes

The number of TUNEL-positive retinal cells in 4-week-old rats (SHR and WKY) was 3.2±2.8 positive cells per retinal section in control WKY rats. This number did not differ significantly from the 2.0±2.7 positive cells per retinal section seen in diabetic WKY rats or the 2.0±2.0 and 0.9±0.8 positive cells per retinal section seen in control and diabetic SHR, respectively. The rates of apoptosis in retinal tissue in 12-week-old rats were also similar among the groups (1.2±1.3 for control WKY versus 1.9±2.7 for diabetic WKY versus 1.6±1.7 control SHR versus 1.1±0.1 for diabetic positive cells/retinal section, p<0.05). Hence, in eight retinal sections, the number of TUNEL-positive cells was 9.6 for control WKY, 15.2 for diabetic WKY, 12.8 for control SHR, and 8.8 for diabetic SHR.

Since diabetes and hypertension influenced the number of BrdU-positive cells only in 12-week-old rats, all subsequent experiments were done only 12-week-old rats.

**Figure 4:**
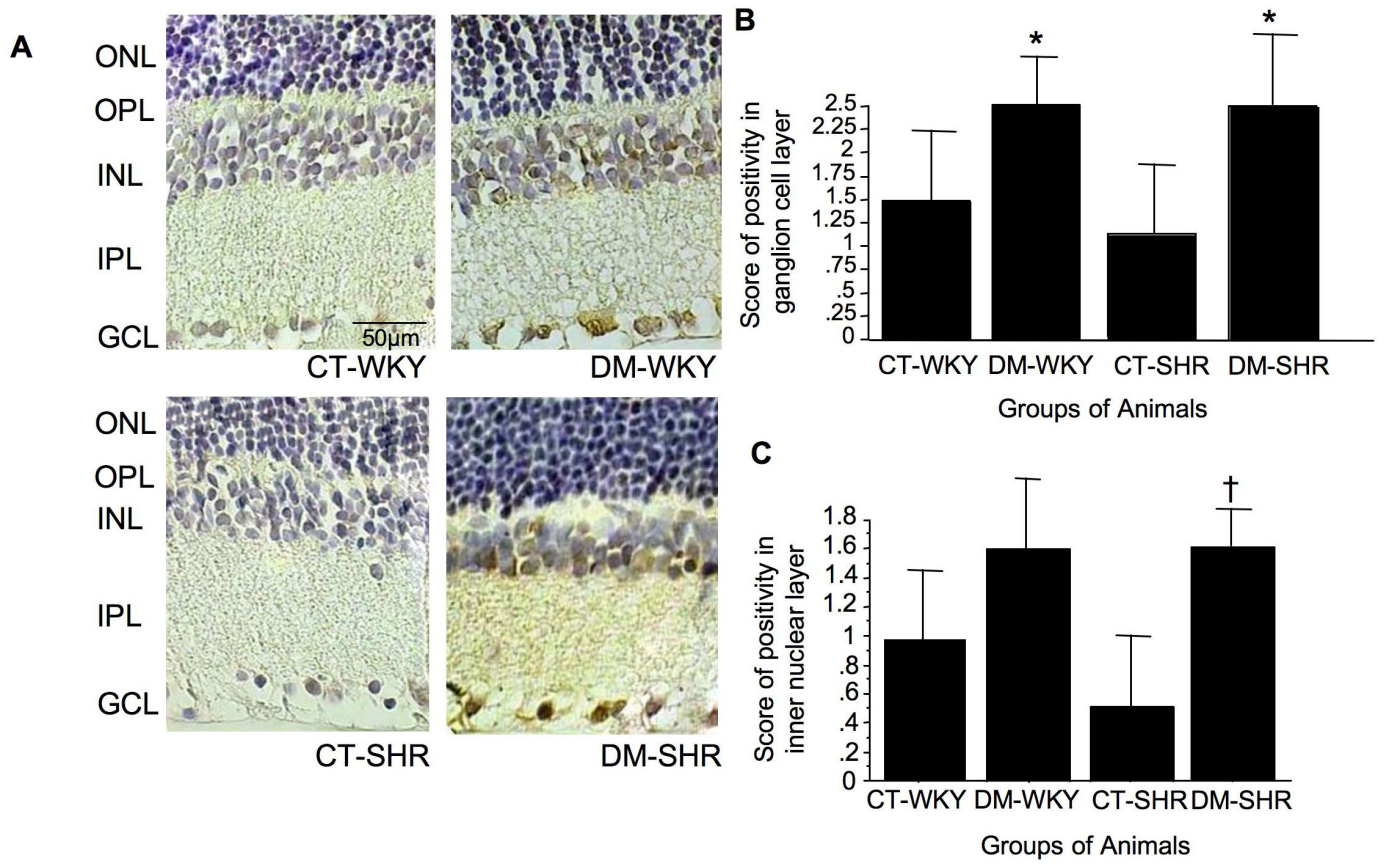
Retinal expression of p27^Kip1^ in retina evaluated through immunohystochemistry assay. In (**A**), we can see a representative immunohistochemical staining for p27^Kip1^ in retina of control and diabetic Wistar Kyoto (WKY) rats and spontaneously hypertensive rats (SHR). The brownish color represents staining for p27^Kip1^, and the blue color represents the staining of retinal tissue with hematoxylin. Original magnification 1000x, the bar in the figure represents a scale of 50µm. The graphs represents the score of positivity of p27^Kip1^ in retinal slides expressed by mean±SD in ganglion cell (**B**) and inner nuclear (**C**) layers. Asterisk (*) is p=0.05 versus CT-WKY and CT-SHR, and dagger (†) is p=0.02 versus CT-SHR.

To investigate a potential role for cell cycle regulatory proteins in the reduction in BrdU-positive retinal cells in 12-week-old diabetic SHR, we examined the expression of p27^Kip1^. Immunohistochemistry revealed a heterogeneous distribution for p27^Kip1^ in the retinal cell layers, with greater positivity in the ganglion cell layer (Figure 4A). There was a significant increase in p27^Kip1^ protein positivity in this layer in diabetic rats (1.5±1 in control WKY versus 2.5±0.5 in diabetic WKY, and 1.5±1.5 in control SHR versus 2.5±1 in diabetic SHR positive score, p=0.05; Figure 4B). Interestingly, there was a significant increase in p27^Kip1^ protein in the inner nuclear layer only in diabetic SHR rats (0.7±0.6 versus 1.6±0.4 positive score for control versus diabetic SHR, p=0.02; Figure 4C). The increase in p27^Kip1^ expression in diabetic WKY, was not significant (0.9±0.5 and 1.6±0.5 positive score for normal and diabetic WKY; Figure 4C). The accumulation of p27^Kip1^ in retinal tissue is accompanied by increased fibronectin expression in diabetic hypertensive rats.

**Figure 5:**
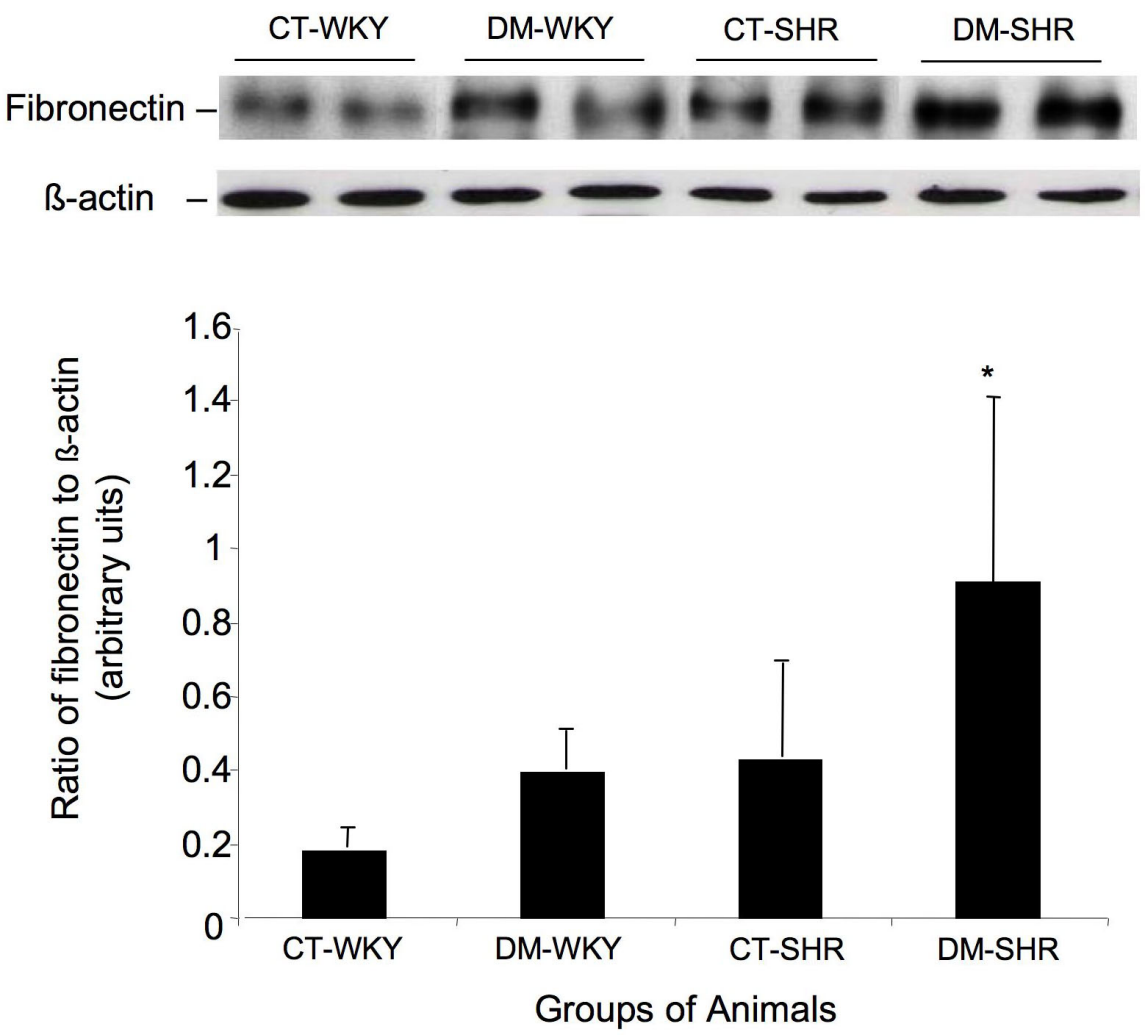
Western blot assay to access the retinal expression of fibronectin. **A:** The membranes were incubated with antibody against glial fibrillar acidic protein in total retinal lysates. **B:** Band densities (ratio of fibronectin to β actin) expressed in arbitrary densitometric units. The columns are the mean±SD of five independent experiments.

To identify factors potentially involved in the pathogenesis of diabetic retinopathy, we examined the retinal expression of fibronectin, an extracellular matrix component associated with functional properties of the inner blood-retinal barrier. Western blot analysis of total retinal lysates revealed a significant increase in fibronectin expression in diabetic SHR compared with control SHR (p=0.04) or diabetic (p=0.03) and control WKY (p=0.009; Figure 5). The enhanced expression of p27^Kip1^ and fibronectin reflected a generalized response to hyperglycemia in target organs such as the kidney. This is the first demonstration of an association between increased expression of p27^Kip1^ and fibronectin accumulation in retinal tissue, and could contribute to thickening of the retinal capillary basement membrane seen in diabetic retina.

### The expression of retinal GFAP is unaltered in SHR or WKY rats

**Figure 6:**
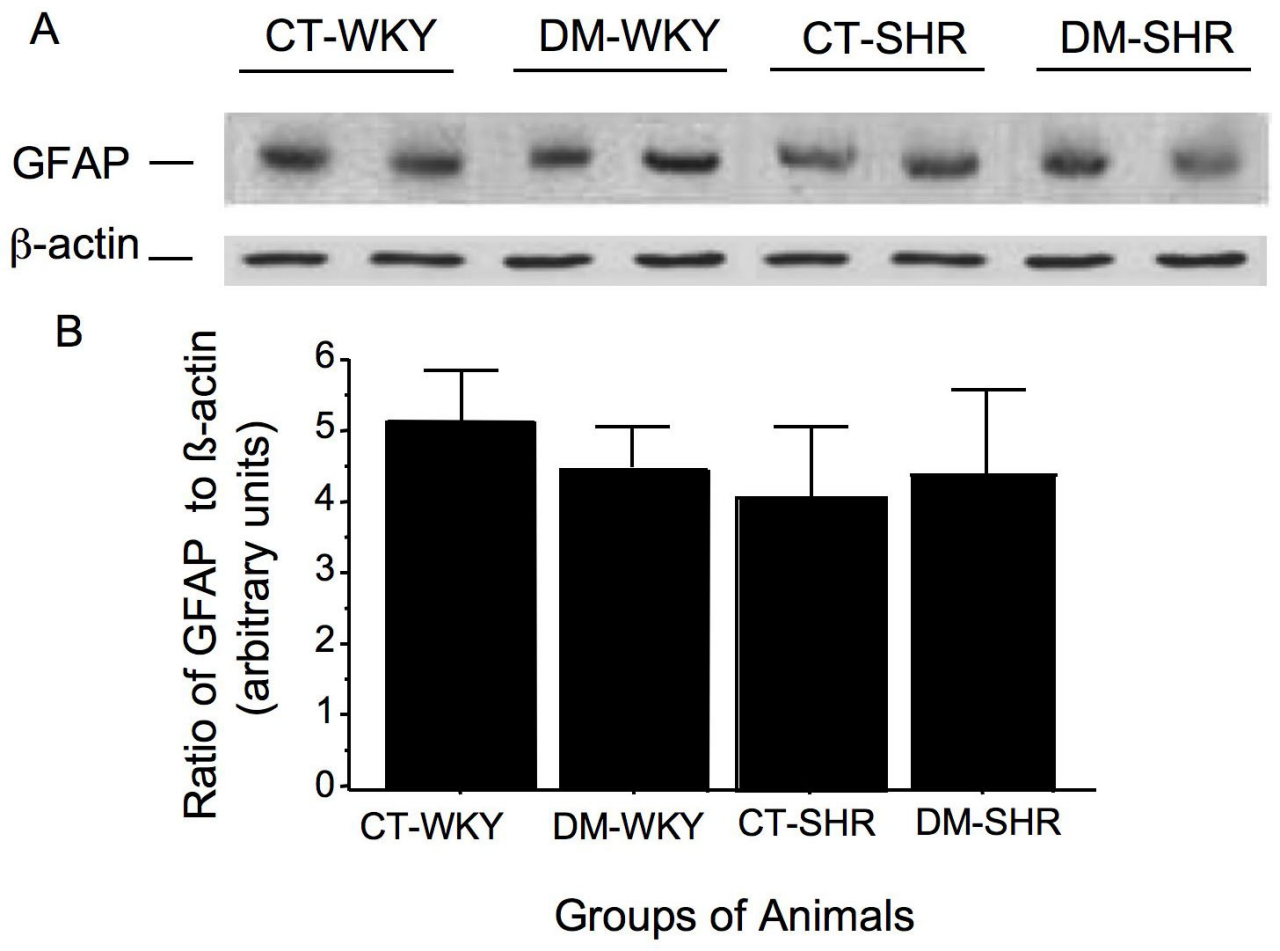
Retinal expression of glial fibrillar acidic protein assayed by western blot. **A:** The membranes were incubated with antibody against glial fibrillar acidic protein (GFAP) in total retinal lysates. The columns represent the band densities (ratio of GFAP to β-actin) expressed in arbitrary densitometric units. Mean±SD of five independent experiments. **B:** Band densities (ratio of GFAP to β-actin) expressed in arbitrary densitometric units. The used symbols are CT-WKY for control WKY, DM-WKY for diabetic WKY, CT-SHR for control SHR and DM-SHR for diabetic SHR.

The retinal expression of GFAP in total retinal lysates, as evaluated by western blotting, was used as an indicator of glial cell reactivity. This expression did not differ between 345 control SHR (5.2±1.4 arbitrary units ) and WKY rats (4.5±0.5 arbitrary units), nor was it significantly altered by 15 days of diabetes mellitus (4.5±0.5 for diabetic WKY and 4.4±0.8 arbitrary units for diabetic SHR; Figure 6)

### VEGF expression is enhanced in diabetic SHR after 15 days of diabetes mellitus

**Figure 7:**
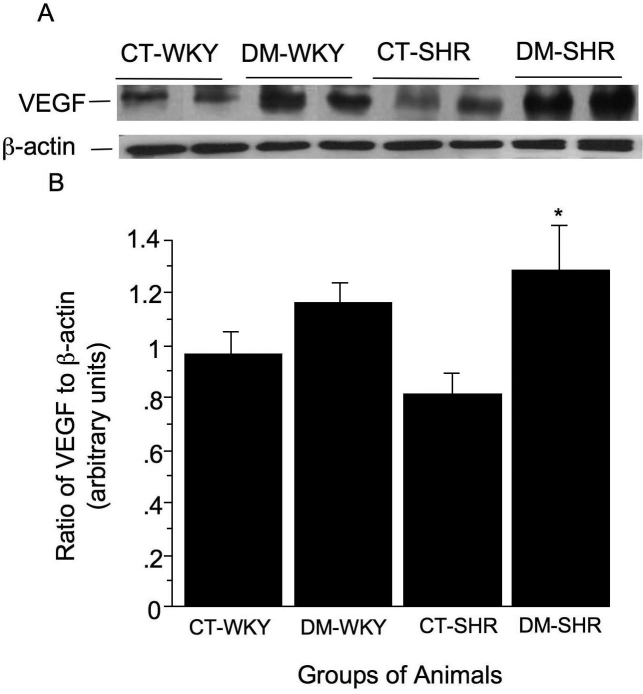
Retinal expression of vascular endothelial growth factor (VEGF) was accessed by western blot assay. In A, the membranes were incubated with antibody against VEGF in total retinal lysates. B: The graph display the band densities (ratio of VEGF to β actin) expressed in arbitrary densitometric units. The columns are the mean±SD of four experiments. The used symbols are CT-WKY for control WKY, DM-WKY for diabetic WKY, CT-SHR for control SHR and DM-SHR for diabetic SHR. Asterisk (*) is p=0.02 versus other groups.

There was a significant increase in the retinal expression of VEGF only in diabetic SHR (0.9±0.1 arbitrary units versus 1.1±0.1 arbitrary units for diabetic WKY and 0.8±0.1 arbitrary units for control SHR and 1.3±0.2 arbitrary units for diabetic SHR, p=0.02) (Figure 7).

### Blood-retinal barrier breakdown occurs only in diabetic hypertensive rats

**Figure 8:**
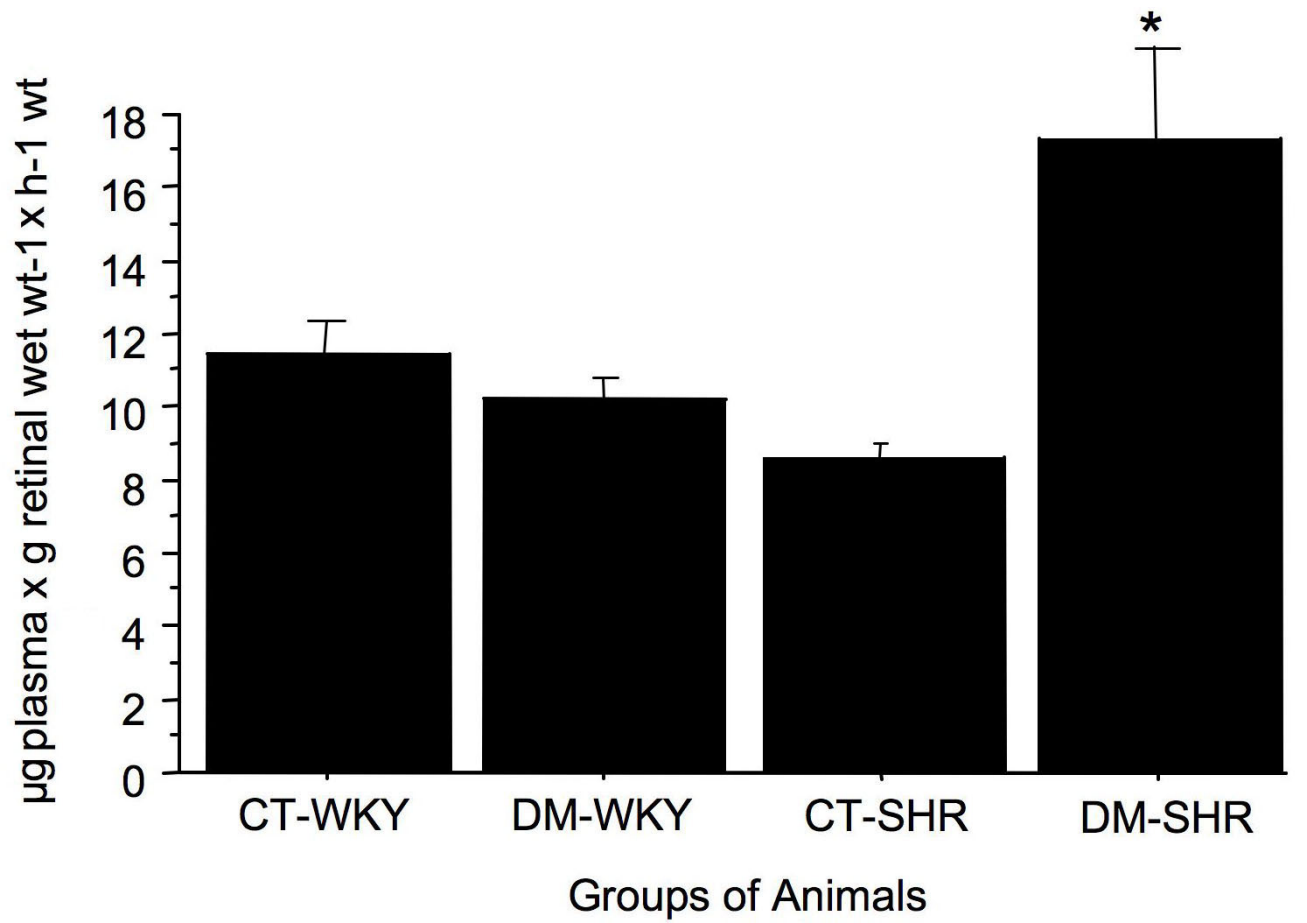
Retinal capillary permeability assessed by the Evans blue method in control and diabetic spontaneously hypertensive rats (SHR) and Wistar Kyoto (WKY) rats. The permeability was expressed in µl of plasma × g retinal wet wt^−1^ × h^−1^ wt. The results are expressed as the mean±SD. The following abbreviations are in effect: control WKY (CT-WKY), diabetic WKY (DM-WKY), control SHR (CT-SHR), and diabetic SHR (DM-SHR).

The extent of blood-retinal barrier breakdown, estimated by the extravasation of Evans blue dye, was similar in control and diabetic WKY rats (11.4±1.5 for control WKY and 10.3±1.3 μg plasma x g retinal wet wt^-1^ x h^-1^ for diabetic WKY; n=5 rats). In contrast, there was a significant increase in retinal capillary permeability in diabetic SHR compared with control SHR (8.6±0.7 for control SHR and 17.4±4.6 for diabetic SHR μg plasma x retinal wet wt^-1^xh^-1^; p=0.01, n=5 rats; Figure 8).

## Discussion

The results of this study show that the adult rat retina contains a small number of BrdU-positive cells that also express glial, neural, and endothelial progenitor cell markers. The number of these cells was higher in hypertensive rats but was markedly reduced by the concomitant presence of hypertension and diabetes. The greater expression of the Cdk inhibitor, p27^Kip1^, probably accounts for the reduction in the number of proliferating cells in diabetic retina. Additionally, diabetic hypertensive rats with a reduction in the number of BrdU-positive cells and enhanced p27^Kip1^ expression also displayed classic characteristics of early diabetic retinal disease: enhanced fibronectin and VEGF expression and greater blood-retinal barrier breakdown. These observations—i.e., cell cycle withdrawal and enhanced production of extracellular matrix and VEGF expression—could provide a basis for a new understanding of the mechanisms involved in the pathogenesis of diabetic retinopathy.

It is known that SHR rats display innate abnormal endothelial function associated with eNOS compensatory activity and increased nitric oxide production [35]. Evidence is accumulating that implicates oxygen free radical formation and N-methyl-D-aspartate (NMDA)-receptor-mediated toxicity in the pathophysiology of ischemic retinal injury [36,37]. These two mechanisms are linked by nitric oxide. NMDA-receptor activation generates nitric oxide, which reacts with superoxide to form toxic species such as peroxynitrite. Neuropeptide Y (NPY) is a 36 amino acid peptide widely present in the central nervous system including the retina. NPY was recently found to stimulate retinal neural cell proliferation mediated through nitric oxide-cyclic guanosine monophosphate (GMP) and extracellular signal-regulated kinases (ERK) 1/2 pathways [38]. These results suggest that the increased retinal cell proliferation observed in SHR rats may be due to activation of NPY through nitric oxide-cyclic GMP and ERK 1/2 pathways. Previous data from other cell types of SHR also demonstrated that mesangial cells have a higher proliferation rate in vitro than control WKY mesangial cells [39], suggesting that the genetics of hypertension may contribute to this phenotype.

The changes described for diabetic hypertensive SHR were not associated with the toxicity of streptozotocin for two reasons: 1) they occurred in the retina of diabetic hypertensive SHR but not in normotensive diabetic WKY rats; and 2) they were absent in diabetic WKY and SHR rats rendered diabetic at four weeks of age. These changes are also not attributable to differences in metabolic control, because the blood glucose levels were similar in both diabetic groups.

The presence of neuronal progenitor cells in human retina has recently been demonstrated. It was suggested that these cells may potentially replace neurons and photoreceptors [40]. The BrdU-positive retinal cells seen in the present study were not completely characterized. However, their proliferative capacity and their colocalization with antigens of neural, glial, and endothelial origin suggest multipotent properties—i.e., they may represent progenitor cells.

Not all dividing cells in the retina are progenitors. Numerous studies have sought to identify neural progenitor cells. The intermediate filament protein nestin has a widespread, early expression in the developing retina and central nervous system and is one of the best-characterized protein markers for immature neural cells [41,42]. The coexpression of nestin with other developmental markers, such as the incorporation of BrdU (such as that seen in the present study) strongly suggests that the cells involved are immature. In addition to nestin, we also observed immunostaining for PKC-α (a bipolar–amacrine cell marker) in BrdU-positive cells in the retina. The coexpression of BrdU and nestin or BrdU and PKC-α was also seen.

The cyclin kinase inhibitor p27^Kip1^ may be involved in reducing the number of proliferating retinal cells. Levine et al. [28] suggested that p27^Kip1^ is part of the molecular mechanism that controls the decision of multipotency central nervous system progenitor cells to withdraw from the cell cycle. These authors also proposed that postmitotic Müller glia have a novel, intrinsic requirement for p27^Kip1^ to maintain their differentiated state. The heterogeneous distribution of p27^Kip 1^ seen in retinal tissue, with greater expression in the inner nuclear layer only in diabetic SHR, may contribute to the different number of replicating cells in this group. Further clarification of the other mechanisms involved in what prompts a replicating retinal cell to withdraw or continue in the cycle when in the presence of diabetes and hypertension is beyond the scope of this study and requires a new experimental design.

In the present study, we did not detected an enhanced apoptotic rate in retina from normotensive diabetic WKY and hypertensive diabetic SHR rats, as reported by others. Thus, for example, Gastinger et al. [43] reported a significant increase in the apoptotic rate in retina from diabetic rats, based on the number of TUNEL-positive nuclei seen in whole-mounted retinas from Sprague-Dawley rats after two weeks of streptozotocin-induced diabetes. The discrepancy between our findings and the latter study may be related to differences in rat strain and in the use of whole-mounted retina compared to retinal cross-sections. In our study, the retinas of diabetic=WKY and SHR rats after 12 weeks of diabetes showed a significant increase in the number of TUNEL-positive cells in the diabetic groups, particularly in diabetic SHR rats (p=0,0003; unpublished).

Increased extracellular matrix protein production leading to structural abnormalities is a hallmark of diabetic microangiopathy and has been demonstrated in all target organs of diabetic complications, including retina, kidney, and heart [44,45]. Enhanced extracellular matrix protein synthesis is instrumental in thickening of the basement membrane [16,46]. Fibronectin is a major extracellular matrix component but its overproduction may decrease the motility and replication of many cells [46]. As revealed in the present study, retinal tissue from diabetic WKY rats and nondiabetic SHR showed enhanced fibronectin expression in total retinal lysates, although this expression was not significantly different from that in control WKY rats.

The accumulation of fibronectin in retinal tissue is a classic finding encountered early in the pathogenesis of diabetic retinal disease and in the microcirculation in models of hypertension [47-49]. Several studies have described the accumulation of extracellular matrix in target organs of hypertensive rats. In stroke-prone SHR there is enhanced accumulation of extracellular matrix proteins in the cerebral vasculature [47,48]. Agabiti-Rosei demonstrated that structural and functional changes in the microcirculation during arterial hypertension, e.g., remodeling of the extracellular matrix and accumulation of collagen and fibronectin, were associated with several neurohumoral and hormonal factors [49]. Hence, the similar extracellular protein accumulation seen here between diabetic WKY rats and nondiabetic SHR may be the result of either diabetes or hypertension alone.

The induction of diabetes in SHR leads to a reduction in renal cell replication with concomitant overexpression of p27^Kip1^ [14]. p27^Kip1^-deficient mice do not develop the classic features of diabetic nephropathy such as renal hypertrophy, glomerular expression of fibronectin, and albuminuria, all of which are marked in wild-type mice [50]. This finding suggests that modulation of p27^Kip1^ function may ameliorate diabetic nephropathy. The effect of a reduction in p27^Kip1^ expression on diabetic retinopathy remains to be elucidated.

In conclusion, the combination of genetic hypertension and experimental diabetes markedly reduced retinal cell proliferation. This reduction was associated with enhanced p27^Kip1^, fibronectin, and VEGF retinal expressions and greater blood-retinal barrier breakdown. Additional studies are required to clarify the mechanisms by which these cellular changes contribute to the structural abnormalities associated with the early pathogenesis of diabetic retinopathy.
